# Metabolic and protein interaction sub-networks controlling the proliferation rate of cancer cells and their impact on patient survival

**DOI:** 10.1038/srep03041

**Published:** 2013-10-24

**Authors:** Amir Feizi, Sergio Bordel

**Affiliations:** 1 Department of Chemical and Biological Engineering, Kemivägen 10, Chalmers University of Technology, SE412 96 Gothenburg, Sweden

## Abstract

Cancer cells can have a broad scope of proliferation rates. Here we aim to identify the molecular mechanisms that allow some cancer cell lines to grow up to 4 times faster than other cell lines. The correlation of gene expression profiles with the growth rate in 60 different cell lines has been analyzed using several genome-scale biological networks and new algorithms. New possible regulatory feedback loops have been suggested and the known roles of several cell cycle related transcription factors have been confirmed. Over 100 growth-correlated metabolic sub-networks have been identified, suggesting a key role of simultaneous lipid synthesis and degradation in the energy supply of the cancer cells growth. Many metabolic sub-networks involved in cell line proliferation appeared also to correlate negatively with the survival expectancy of colon cancer patients.

Cancer metabolism has been the object of a substantial amount of interest during the last years^1^,^2^. However, most of the attention is focused on a small set of metabolic features, such as the well-known Warburg effect^3^, the catabolism of glutamine^4^, the synthesis of fatty acids^5^,^6^ or the correlation of glycine uptake with cell growth rate^7^. Some cancer associated metabolic features are at the basis of anticancer therapies; polyamine metabolism^8^,^9^, biosynthesis of geranylgeranyl diphosphate^10^ and biosynthesis of prostaglandin E2^11^, are some relevant examples.

Genome-scale metabolic models^12^ are promising tools for the identification of new metabolic drug targets^13^,^14^. The recently published consensus human metabolic model Recon2^15^, and the last version of the HMR database^16^, are comprehensive high quality models of human metabolism. Recon2 contains 7440 reactions (including transport steps) and 1789 metabolic genes. The HMR database contains 8100 reactions and 3668 metabolic genes. Among those genes, 1647 are shared between both models, 147 are unique to Recon2 and 2021 are unique to HMR. Protein interaction networks, are also available^17^ to be used as tools for the contextualized analysis of high throughput experimental data.

This paper is aimed at identifying metabolic sub-networks, as well as regulatory mechanisms and protein interaction sub-networks that control the growth rate of cancer cells. A previous study^7^ showed that both the glycine uptake rate and the expression level of the gene SHMT2, involved in glycine synthesis from serine, are positively correlated with the growth rate across the NCI-60 cell panel^18^. This suggested that SHMT2 is a suitable target for decreasing the proliferation rate of cancer cells. This hypothesis was proven by silencing SHMT2 in HeLa cells, which led to a strong increase of the cell doubling time and the associated decrease in proliferation rate. Our work is based on the same assumption as Jain and co-workers^7^, namely that genes whose expression shows a significant positive correlation with cell growth (across the NCI-60 panel) are potential targets against cell proliferation (even in cell lines not belonging to the NCI-60 panel such as HeLa cells).

Genome-scale metabolic networks or protein interaction networks can be used for a contextualized data analysis. For example if the expression levels of several metabolic genes linked to reactions that are stoichiometricaly coupled between each other (for example reactions in a linear metabolic pathway), are positively correlated with the growth rate, it is likely that the activity of the pathway has a causal relationship with the cell growth rate. This is not the case if only a single gene linked to this pathway shows a significant correlation.

Another advantage of using genome-scale biological networks is the fact that instead of single genes it allows identifying sub-networks. This helps to choose combinations of targets that would disable the function of one or several of the relevant sub-networks and lead to a synergistic effect against cancer proliferation, this phenomenon is known as synthetic lethality^1^,^13^. In this way it will be possible to design combinatorial drug treatments, which can be tested experimentally. The NCI-60 panel has been used to test the effects of thousands of different drugs^18^, however, testing all the possible combinations of several drugs, would result in a combinatorial explosion. Computational approaches based on the mechanistic information contained in genome-scale biological networks are necessary in order to focus the experimental efforts on a smaller set of promising combinatorial treatments.

A new algorithm to identify metabolic sub-networks showing a correlated activity with the growth rate has been developed. Contrarily to protein interaction networks, metabolic networks are topologically Petri nets and not graphs. The proximity between two reactions cannot be just defined by the existence of shared metabolites. If two reactions are stoichiometrically coupled (e.g. their fluxes are fully correlated) they should be considered as neighbours even if they do not share metabolites. A new algorithm taking account of this fact has been developed. The details of the algorithm are presented in the supplementary material. The number and complexity of the metabolic sub-networks identified by the new algorithm, reveals that cancer metabolism goes well beyond the few features that are normally the main object of attention in the literature^2^.

There is an underlying assumption in using correlations observed in the NCI-60 panel to extract general conclusions about cancer metabolism. This assumption is that although cancer cells are heterogeneous even within the same tumor^19^, the fact that all the cancer cells need to overcome certain constraints in order to proliferate gives them certain common hallmarks^20^. In order to test the suitability of the mentioned extrapolation, we used a study comparing the gene expression of 145 colon cancer patients who survived more than 5 years and 85 patients who died within 5 years^21^. These data were accessed through the G-DOC database (https://gdoc.georgetown.edu). This study was chosen based on the high number of patients, which allows increasing the statistical power of the test used to identify differentially expressed genes. The newly developed algorithm was used to identify metabolic sub-networks showing higher activity in the group of patients who died in the first 5 years. The results evidenced that many of the metabolic processes involved in the faster proliferation of cancer cell lines are also involved in higher mortality of colon cancer patients. This confirms the suitability of targeting these metabolic processes.

## Results

We present here the main results and conclusions of several forms of integrated analysis of gene-expression data. Our main focus is cell metabolism and the integrated analysis of gene expression patterns within the context of genome-scale metabolic networks; however, in order to put our results in the context of the regulatory mechanisms triggering cell growth, we start by identifying transcription factors and protein interaction sub-networks that show an increased activity in cells with higher proliferation rates.

### Correlation of gene expression with cell line growth

**Figure 1:**
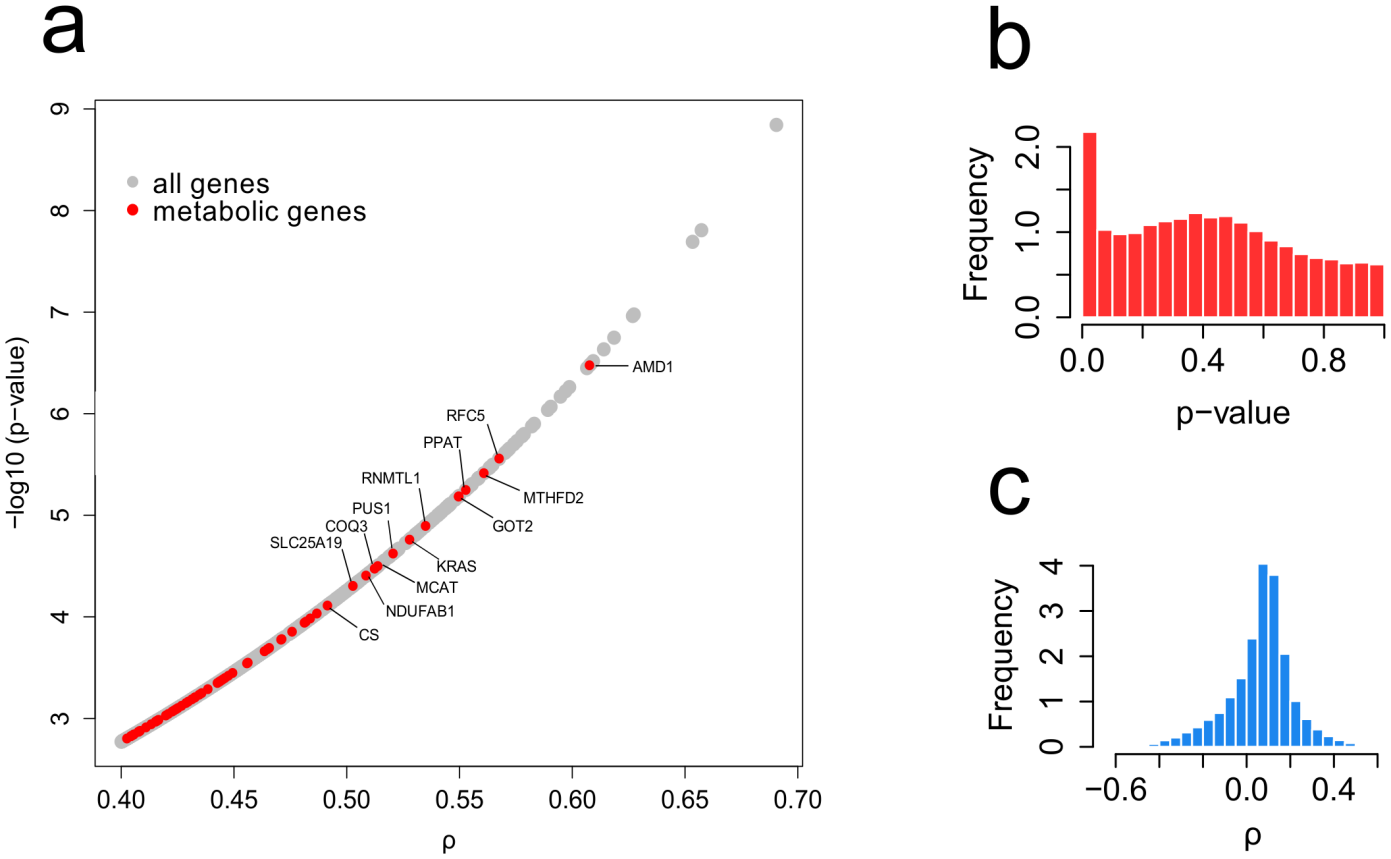
Panel a shows the Spearman correlation coefficients and associated p-values of some of the metabolic genes that show the most significant correlations. The panels b and c show the distributions of Spearman correlation coefficients and p-values. The higher frequency of low p-values shows that there are more growth correlated genes than those that could be expected as an artifact of multiple testing (which would correspond to a flat distribution of p-values).

The normalized HG-U133 Plus 2.0 and the HG-U95 microarrays available at Cell-Miner (http://discover.nci.nih.gov) were used as input data to our analysis. We started computing a Spearman correlation coefficient with the cell growth rate for each probe in the mentioned microarrays. The histograms of correlation coefficients and p-values for the HG-U133 Plus 2.0 microarray are shown in figure 1, together with the p-values and correlation coefficients of some of the most correlated metabolic genes. Using the obtained p-values (see supplementary material for the detailed method) we selected a set of positively correlated probes with a false discovery rate of 0.05 for each of the arrays. There were 318 genes (identified by their Entrez ids) that appeared to have a significant positive correlation with the growth rate in both arrays (with a 0.05 false discovery rate). The Entrez ids were mapped into 288 Ensembl ids. We also identified 128 genes showing significant negative correlation with the growth rate (supplementary dataset S1). These gene sets were used to identify enriched transcription factor binding motifs. Among the genes positively correlated with the growth rate there are 10 genes (supplementary dataset S1) that had been previously identified (using gene silencing) as highly essential for proliferation in a broad set of cancer cell lines^22^. Some of them are NDUFAB1, which codes a sub unit of the NADH dehydrogenase transforming ubiquinone into ubiquinol; KARS, which codes a lysyl-tRNA synthetase; RPL6, coding a ribosomal protein; HNRNPM, coding a heterogeneous nuclear ribonucleoprotein involved in mRNA preprocessing etc. Among the 288 genes showing the most significant correlation with the growth rate, 72 were metabolic genes included in the HMR database^16^. Only 35 of them were present in Recon2^23^, which has lower gene coverage than the HMR database.

### Transcription factors controlling cancer growth rate

**Figure 2:**
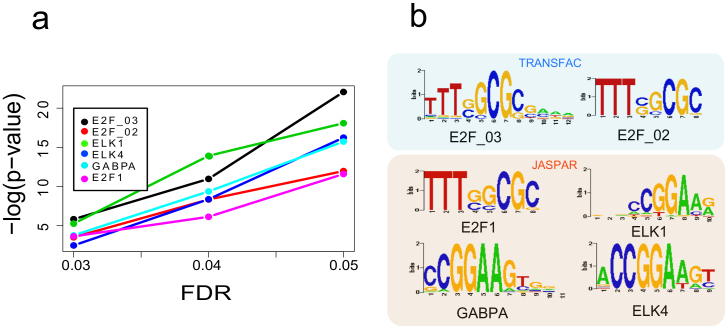
Enrichment p-values for several transcription factor binding motifs in the JASPAR and TRANSFAC databases. The p-values have been computed for 3 different sets of top correlated genes (defined by false discovery rates of 0.01, 0.03 and 0.05).

In order to identify the mechanisms controlling the proliferation rate of cancer cell lines at the transcriptional level, the set of genes showing significant positive correlation with the growth rate (with a 0.05 false discovery rate) in both microarrays, was used to identify enriched transcription factor binding motifs. We used Pscan^24^ to identify the motifs. Pscan identified 6 position specific weight matrixes with p-values lower than 1e^−10^, 4 of them from the JASPAR database^25^ and 2 from the TRANSFAC database^26^. In order to check for consistency, we repeated the analysis using gene sets with false discovery rates of 0.03 and 0.01 respectively. The p-values decreased with the number of tested genes but the same 6 motifs were always the most significantly enriched (figure 2).

The identified binding motifs from JASPAR corresponded to ELK1, ELK4, GABPA and E2F1. The two TRANSFAC motifs were E2F_02 and E2F_03. These motifs are potential targets of the E2F family of transcription factors. The lists of regulated genes and the position of the binding motifs in their promoters are reported in the supplementary dataset S2.

The same kind of analysis was performed for the set of genes that showed significant negative correlations with the growth rate (with 0.05 false discovery rate) in both microarrays. This led to the identification of Egr1 and SP1 with p-values of 2.7e-9 and 2.5e-8 respectively.

### Identification of growth correlated protein interaction sub-networks

**Figure 3:**
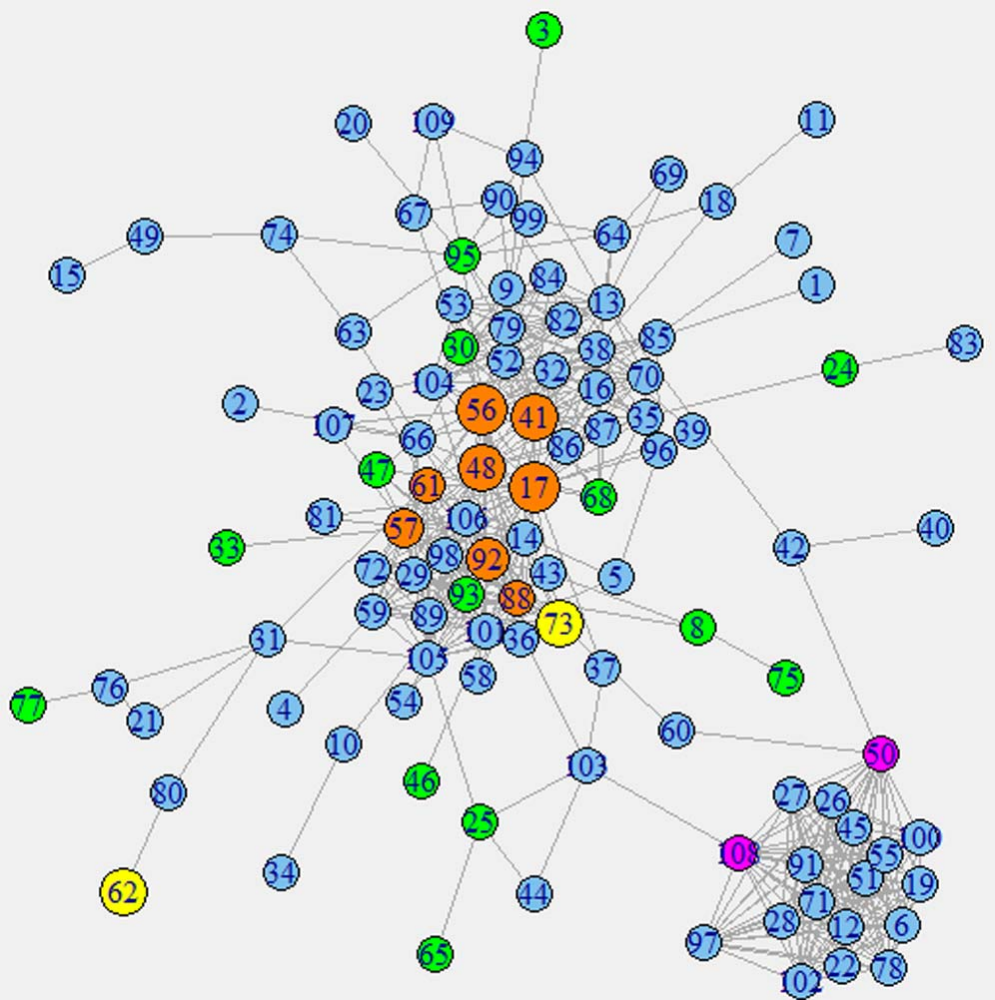
Protein interaction graph corresponding to the growth correlated genes. The numbers in each node correspond to the identifiers in supplementary file S1. The yellow nodes correspond to proteins involved in regulatory feedbacks such as SKP2 (node 62) and p130 (node 73). We can see that p130 interacts with 17 other growth correlated proteins, which correspond to cytoplasmic ribosomal proteins and heterogeneous ribonucleoprotein particles (hnRNPs). The whole list of genes in the network can be found in the supplementary file S1. The orange nodes correspond to the most connected hubs of the main cluster. They are ribosomal proteins such as RPS9, RPS6, RPL4 or RPL13A. The green nodes correspond to proteins that also have a metabolic activity and the purple nodes are the most connected hubs in the second cluster, which correspond to mitochondrial ribosomal proteins.

Among the 288 genes that showed significant positive correlation with the growth rate (with 0.05 false discovery rate) in both microarray platforms, 124 showed at least one protein interaction with another of the genes. Among those genes, 109 formed a connected sub-network (figure 3). This network is characterized by two clear clusters of proteins tightly connected between each other. The smaller cluster corresponds to mitochondrial ribosomal proteins, while the bigger cluster includes mostly cytoplasmic ribosomal proteins and heterogeneous ribonucleoprotein particles (hnRNPs), which are involved in the splicing and preprocessing of RNAs. It is well known that many ribosomal proteins belonging to both ribosomal subunits are overexpressed in cancer cell lines and tumors^27^, which also leads to morphological changes in the nucleolus^28^. Higher expression of the protein translational machinery, including the preprocessing of mRNAs, has a very straightforward quantitative relationship with higher proliferation rates. However there are also qualitative features of cancer that can be explained by higher expression of these components. In particular hnRNPA1, whose expression level is controlled by E2F1 and positively correlated with the growth rate, is involved in the alternative splicing of PKM (pyruvate kinase)^29^. The increased activity of hnRNPA1 results in the formation of the splicing variant PKM2 instead of PKM1. This is believed to be the cause of the Warburg effect^29^.

### Metabolic sub-networks controlling cell growth rate

We have developed a new algorithm for the integration of gene-expression data with genome-scale metabolic networks. Metabolic networks are topologically Petri nets and transforming them into graphs (in which the adjacency between two reactions is defined by sharing metabolites) results necessarily in a loss of information and a misrepresentation of their structure. Here we define a measurement of the functional similarity between metabolic reactions. This measurement is based on projecting the vector representing each reaction into the kernel of the stoichiometric matrix of the metabolic network and computing the angles between the projections of different reactions. In this way, reactions that are fully stoichiometrically coupled (even if they do not share any metabolite) have a zero angle between them (a detailed description of the methodology is presented in the supplementary material). This measurement of similarity between reactions combined with the statistical significance associated to each reaction (what we call h-value; see supplementary methods), allows identifying reaction clusters showing significant positive correlation with the growth rate. In a second step, all the remaining reactions stoichiometrically coupled to each sub-set are added in order to provide a connected network as an output. We used both the Recon2 genome-scale metabolic model and the HMR database. There is a substantial overlap between the metabolic processes identified using both models, but the sub-networks identified with the HMR database include more reactions, due to the fact that HMR has a more comprehensive gene annotation. The top 100 sub-networks (by number of reactions) obtained using HMR and the top 50 using Recon2 are presented in the supplementary datasets S3, S4, S5 and S6. These files contain the stoichiometry of each of the reactions in the identified networks (with the metabolites named the same way as in HMR and Recon2 respectively) followed by the list of genes associated to each reaction.

Some of the sub-networks identified by the algorithm involve the synthesis and degradation of keratan sulfate I, the synthesis of keratan sulfate II and the synthesis of heparan sulfate proteoglycan. For example the biosynthesis of keratan sulfate I consists on a linear pathway of 37 reactions among which 25 show correlation with the growth rate at the expression level. Keratan sulfate and heparan sulfate are known to play a key role in cell proliferation and metastasis^30^.

**Figure 4:**
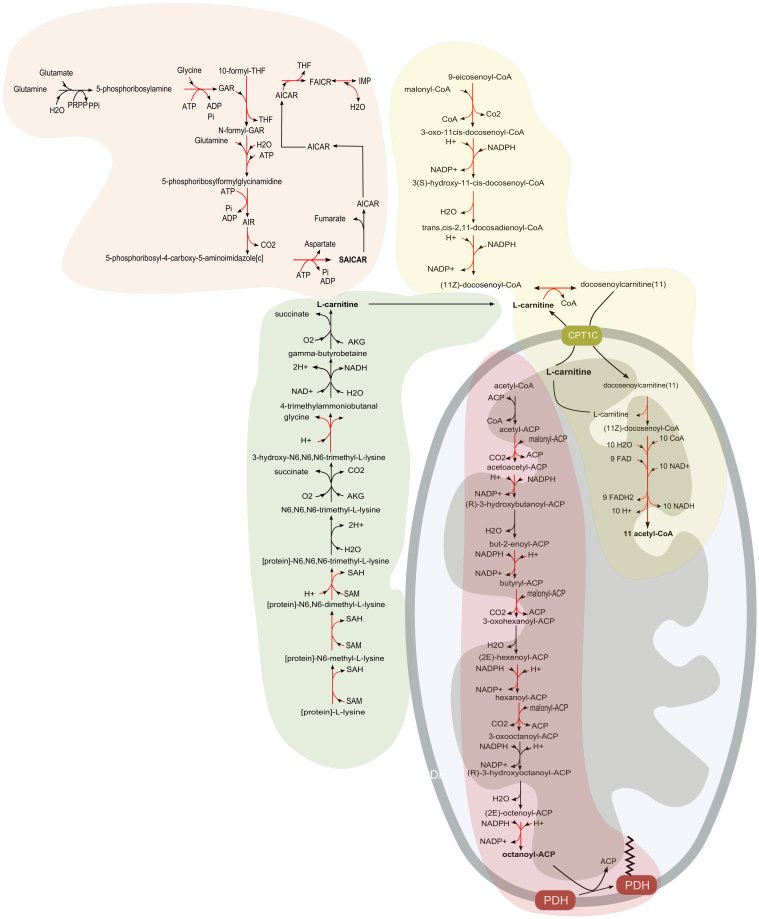
Examples of 4 growth correlated sub-networks. The reactions marked in red show positive transcriptional correlation with the growth rate. The three sub-networks involved in lipid biosynthesis and degradation re-appear among the metabolic sub-networks found in the patient mortality analysis.

A very interesting sub-network (network 13 in the supplementary files S3 and S4) involves the biosynthesis of SAICAR (figure 4), which has been recently reported to stimulate the activity of PKM2 promoting cancer survival in glucose-limited conditions^31^.

The vast majority of the identified sub-networks are related to lipid metabolism, which is consistent with previous knowledge. The higher expression in cancer cells of the FAS I complex (fatty acid synthase) is well known, however the function of the identified sub-networks seems to go well beyond the synthesis of lipids as biomass building blocks. The sub-network analysis identified the synthesis of several acyl-ACPs (catalyzed by the complex FAS II) such as octanoyl-ACP (figure 4), which is required for mitochondrial protein lipoylation. In particular, lipoylation of the E2 subunit of PDH is required for the conversion of pyruvate to acetyl-CoA^32^, which leads to a positive feedback in the de novo synthesis of fatty acids. The synthesis of octanoyl-ACP is known to occur in the mitochondria; however the HMR database presents it in the cytosol. The mentioned mistake should be corrected in future versions of this human genome-scale metabolic model.

Several sub-networks indicate not only an increased rate of fatty acid synthesis but also an increased degradation of very diverse fatty acids. This is in agreement with previous observations^33^, which suggested the coexistence of high levels of fatty acid synthesis and degradation in some cancer cells. Many of the identified sub-networks consist on the cytosolic modification of fatty acids by acyl-CoA synthetases followed by the mitochondrial β-oxidation or the peroxysomal β-oxidation. The peroxysomal β-oxidation is undergone by long and branched fatty acids such as (2R,6R,10R)-trimethyl-hendecanoyl-CoA (network 6 in the supplementary files S3 and S4). In particular, the enzyme α-methylacyl CoA racemase (AMACR), which catalyzes the racemization of α-methyl and carboxylic branched chain acyl-CoA thioesters (network 6 in S3 and S4) preparing them for catabolism in peroxisomes and mitochondria, has been already observed to be overexpressed in many cancers^34^. The transport of fatty acids from the cytosol to the mitochondrion involves the carnitine shuttle, catalyzed by the enzymes CPT-I and II. The limiting substrate of this process has been reported to be carnitine^35^. Our analysis shows also that the de novo synthesis of carnitine (network 41 in S3 and S4) is positively correlated with the cell growth rate (figure 4), which confirms the key role of mitochondrial fatty acid degradation.

The largest sub-network (network 2 in S3 and S4) groups sets of reactions that have the common characteristic of producing hydrogen peroxide or other compounds inducing oxidative stress and inflammation, which is a very well-known feature of cancer. It is believed that the increased oxidative stress of cancer is associated to an abnormal function of the respiratory chain^36^, however network 2 as well as other sub-networks such as 24 and 25, point to important cytosolic and peroxisomal sources of hydrogen peroxide and other oxidant compounds. This sub-network also includes the transformation of arachidonic acid into several forms of HETE (hydroxyeicosatetraenoic acid), which has been shown to have a strong antiapoptotic effect and promote mitogenesis^37^.

### Impact of metabolism on the survival of colon cancer patients

In order to assess to which extent the identified sub-networks can be extrapolated from a panel of cancer cell lines to in-vivo tumors; we have performed a differential expression analysis of 145 colon cancer patients who survived more than 5 years and 85 patients who died within 5 years^21^. The mentioned study was chosen among those contained in the GDOC database (https://gdoc.georgetown.edu) because it contains the highest number of patients and it allows obtaining the highest statistical confidence for the differential expression of genes between both groups of patients.

A t-test between the two groups of patients was performed and the p-values obtained were used to carry out the same analysis as in the case of growth correlated genes in the NCI-60 panel. In general the statistical significance of differential expression between the patient groups is much lower than the significance of correlation across the NCI-60 panel. Only 8 genes passed a threshold of 0.05 false discovery rate compared with 288 in the previous analysis. However, the metabolic sub-network analysis is still possible because it is not based on a stringent significance cut-off (see supplementary material for a detailed description of the algorithm). The analysis was performed using the HMR database due to its more comprehensive gene annotation.

The identified metabolic sub-networks are presented in the supplementary files S7 and S8. Interestingly 24 out of the larger 50 sub-networks overlap with some the sub-networks identified in the previous analysis (supplementary files S3 and S4). This suggests that many of the metabolic processes controlling the growth rate of cancer cell lines are also playing the same role in colon cancer and have an impact on the survival expectancy of the patients. Lipid metabolism is strongly predominant among the identified metabolic sub-networks.

A good way to compare the results of the two types of analysis is to assess the degree of overlap between the metabolites unique to the top 100 sub-networks identified in each case. In the growth rate correlated sub-networks there are 852 metabolites that appear only within these sub-networks. In the case of the mortality correlated sub-networks there are 332 exclusive metabolites, 162 of these metabolites are in the intersection of both metabolite sets. The total number of metabolites in the model is 5552 and the probability of finding such an overlap randomly is virtually zero (see supplementary material). This suggests that the same metabolic mechanisms responsible for a higher in-vitro growth rate of the cell lines are also responsible for higher growth rate of colon tumors in vivo and the associated higher patient mortality.

The list of overlapping metabolites is provided in the supplementary file S9. Particularly interesting are those metabolites that, after blocking the reactions in which they are involved, result in the degrees of freedom of their sub-networks dropping to zero (in both the growth related and the mortality related sub-networks). The predominant compounds are lipids, but there are also some interesting exceptions such as fructose-2,6-biphosphate. This compound is an allosteric activator of PFK-1. Its role in cancer has been recently reviewed^38^ and the action of the anticancer drug N-bromoacetiletanolamine is based on targeting the enzyme PFK-2, involved in the synthesis and degradation of fructose-2,6-biphosphate.

## Discussion

The transcription factors of the E2F family are known to be key regulators of the cell cycle and are involved in tumorigenesis^39^. E2F1 is active in the late G1 phase and the S phases of the cell cycle and its overexpression is able to drive cells out of quiescence and also confers transforming potential to primary cells^39^. Its oncogenic activity is restricted by the binding of pRB and E2F1 mutants that cannot bind to pRB are potentially responsible for cancer development. E2F1 has been found to be elevated in colon cancers^40^ and the expression levels of genes with the E2F_03 binding motif from TRANSFAC has been shown to be negatively correlated with the survival time of breast cancer patients^41^.

E2F4 binds to the same sequence motifs than E2F1 but acts as a repressor instead of an activator^39^. In cells in a quiescent state, the promoters regulated by the E2F family are occupied by a complex containing E2F4 and p130 among other proteins. This complex is displaced by E2F1 in the mid G1 phase^39^.

ELK1 and ELK4 belong to the ETS family of transcription factors, which are involved in the response to the activation of growth factor receptors via the MAPK signaling pathway. These transcription factors are known to be involved in cancer by promoting not only growth but also invasion and metastasis^42^. Many of the genes positively correlated with the growth rate have binding motifs for both the E2F family and ELK1 or ELK4. This shows the existence of redundancy in the regulatory mechanisms triggering cell proliferation.

GABPA, also known as Nrf2, is a transcription factor involved in adaptation to hypoxia, which is known to have an increased activity in several cancer types^43^.

A literature survey revealed that SP1 is involved in the expression of p21, which arrests cell proliferation by interacting with cyclin-CDK complexes^44^. It is therefore possible that most of the genes showing a negative correlation with the growth rate are just reporting a lower activity of SP1 that results in a lower expression of p21 and lower inhibition of the cell cycle progression.

In order to gain more insights into the regulatory mechanisms, we used a genome-scale protein interaction network^17^, to identify possible interactions between the transcription factors identified using Pscan, and the products of genes showing significant positive correlations with the growth rate. Only two interactions were revealed, the first one occurs between E2F1 and SKP2. The expression level of SKP2 is regulated by E2F1 and shows a positive correlation with the growth rate. The existence of a positive feedback loop involving E2F1 mediated transcription of SKP2 and enhanced transcriptional activity of E2F1 by SKP2 has been experimentally shown in lung tumors^45^. Our analyses suggest that it is a more general feature of fast proliferating cancer cells. The second observed interaction occurs between the transcriptional repressor E2F4 and p130, which is coded by the gene NOLC1. These two proteins are known to be involved in a complex that blocks the binding sites of E2F1 keeping the cells in a quiescent state^39^. NOLC1 is itself transcriptionally regulated by E2F1, which suggests a negative feedback mechanism that moderates the excessive activity of E2F1. The transcriptional level of NOLC1 shows a positive correlation with the growth rate but this higher expression does not seem to be sufficient to arrest the growth rate of the studied cancer cell lines. This could be explained by a lack of activity of the protein p130, which belongs to the retinoblastoma family and is known to be inactivated by phosphorylation.

Both of the proteins that we found to be involved in positive and negative regulatory loops appear also in this connected sub-network. SKP2 interacts only with CDT1, which is a protein involved in DNA replication. SKP2 is involved in the ubiquitination and subsequent degradation of CDT1^46^. It might seem counterintuitive that SKP2 is involved in the degradation of a protein necessary for DNA replication and have a positive correlation with the growth rate. However, the degradation of the protein CDT1 is necessary for the cell to move from the S phase to the G2 phase of the cell cycle. Therefore its faster degradation helps the cell to transit faster from S to G2, while its higher expression level (the expression level of CDT1 is also positively correlated with the growth rate) allows the cell to go faster through the S phase. In contrast, p130 interacts with 17 other proteins, including the most connected hubs of the network. These hubs include the heterogeneous nuclear ribonucleoproteins M (hnRNPM) and also hnRNPA1 (involved in the alternative splicing of PKM). Also ribosomal proteins such as RPS9, RPS6, RPL4 or RPL13A interact with p130. The density of interactions that p130 shows with elements of the RNA processing and the protein translational machinery suggests that it could be interfering with their function and acting as a growth inhibitor also at this level and not only by cooperating with E2F4 to repress gene transcription. This potential growth inhibitory function seems to be unpaired at the protein level in the studied cancer cells despite the fact that the transcription level of p130 shows a significant positive correlation with the growth rate.

**Figure 5:**
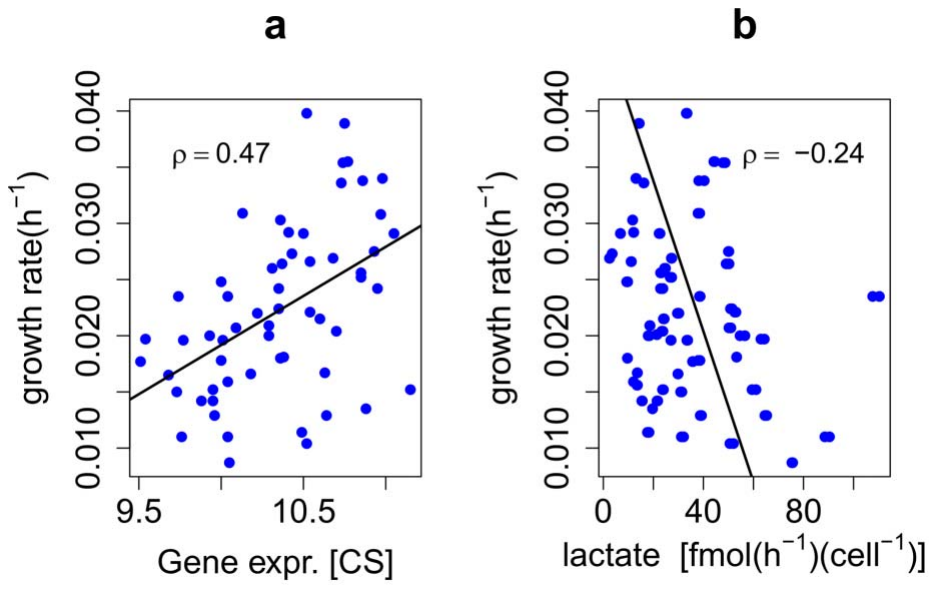
Correlation of cell growth rate with lactate production and gene expression of citrate synthase. The correlation with lactate production is actually negative, which contradicts the hypothesis of glycolysis being the main source of ATP for cancer cells.

**Figure 6:**
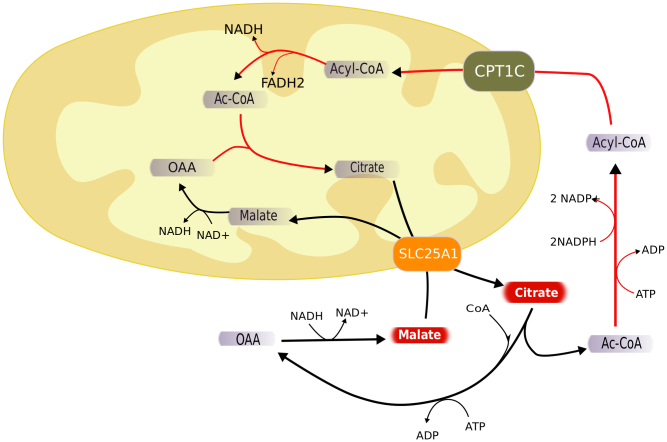
Schematic representation of the hypothesized cycle combining simultaneous fatty acid synthesis and degradation.

The fact that the oxidation of fatty acids, in particular through the mitochondrial β-oxidation, clearly correlates in activity with the growth rate of cancer cells, suggests that lipid oxidation is their main ATP source and not glucose lactic fermentation, known as the Warburg effect. In order to check this hypothesis we computed the Spearman correlation coefficient between the growth rate and the lactate secretion rate reported by Jain and co-workers^7^ for the NCI-60 cell panel. The correlation between growth rate and lactic acid production was slightly negative, with a Spearman correlation coefficient of −0.241 and an associated p-value 0.008, which shows that there is actually a rather significant negative correlation between lactic fermentation and cell growth rate and not a positive correlation, as it would be expected if the glycolysis was the main source of ATP fueling growth. This means that the main source of ATP in cancer cells is still the respiratory chain and according to the results from the integration of gene expression with genome-scale metabolic models, β-oxidation of fatty acids seems to be an important energy source controlling the cell proliferation rate. Another piece of evidence in this direction is the fact that the correlation of the expression level of citrate synthase with the growth rate was positive, with a Spearman correlation coefficient of 0.503 and a p-value of 4.95e-5 (figure 5). A higher expression level of a single gene does not necessary imply a higher metabolic flux, but in this case the positive correlation of L-carnitine biosynthesis as well as fatty acid synthesis and degradation come together to strengthen the hypothesis of a cycle involving simultaneous fatty acid synthesis and degradation (figure 6).

In order to assess if the apparently futile cycle formed by the simultaneous fatty acid synthesis and degradation can constitute an energy source for the cell, we have calculated the global stoichiometric balance of a cycle involving the synthesis of acyl-CoAs from cytosolic acetyl-CoA, the transport of acyl-CoAs to the mitochondria via the carnitine shuttle, the mitochondrial β-oxidation, the transformation of the resulting mitochondrial acetyl-CoA into citrate, the transport of citrate to the cytosol via the citrate-malate antiporter and finally the cytosolic conversion of citrate into acetyl-CoA. The whole cycle consumes two molecules of ATP per unit of cycled acetyl-CoA and results in the consumption of two molecules of cytosolic NADPH and the production of one mitochondrial NADH and one mitochondrial FADH_2_. Also one unit of NADH is consumed in the cytosol and another one is produced in the mitochondria. Therefore the complete cycle results in the reduction of three mitochondrial redox cofactors at the expenses of the oxidation of three cytosolic redox cofactors. The respiratory chain could produce up to 8 ATP molecules from two NADH molecules and one FADH_2_ molecule (assuming a P/O ratio of 3). For a more realistic P/O ratio of 1.5 it could be possible to obtain 4 ATP molecules per cycle, which is still enough to compensate for the loss of 2 ATP molecules necessary to drive the cycle.

The mentioned hypothesis is supported by the fact that the drug etomoxir, an inhibitor of β-oxidation, shows anti-cancer effects^47^ and that expression of carnitine palmitoyltransferase 1C (CPT1C) involved in the carnitine shuttle, promotes cell survival and tumor growth^48^. In a recently published paper^49^ it has been observed a strong cytotoxic effect and high cytosolic lipid accumulation of on Burkitt's lymphoma cells when they are treated with ST1326, an inhibitor of carnitine-palmitoyl transferase 1A (CPT1A).It has also has been shown that pharmacologic inhibition of fatty acid oxidation sensitizes human leukemia cells to apoptosis induction^50^. Other observation pointing in the same direction is the fact that besides lactate, citrate and malate are two of the compounds secreted by all the cells in the NCI-60 panel in higher amounts^7^. This is consistent with high cytosolic concentrations of both compounds, necessary to drive the cytosolic synthesis of acetyl-CoA and the citrate-malate antiporter.

The impact of fatty acid oxidation on patient survival has been previously shown in breast cancer^51^. It was also shown that fatty acid oxidation is triggered by a higher activity of the regulator PML.

**Figure 7:**
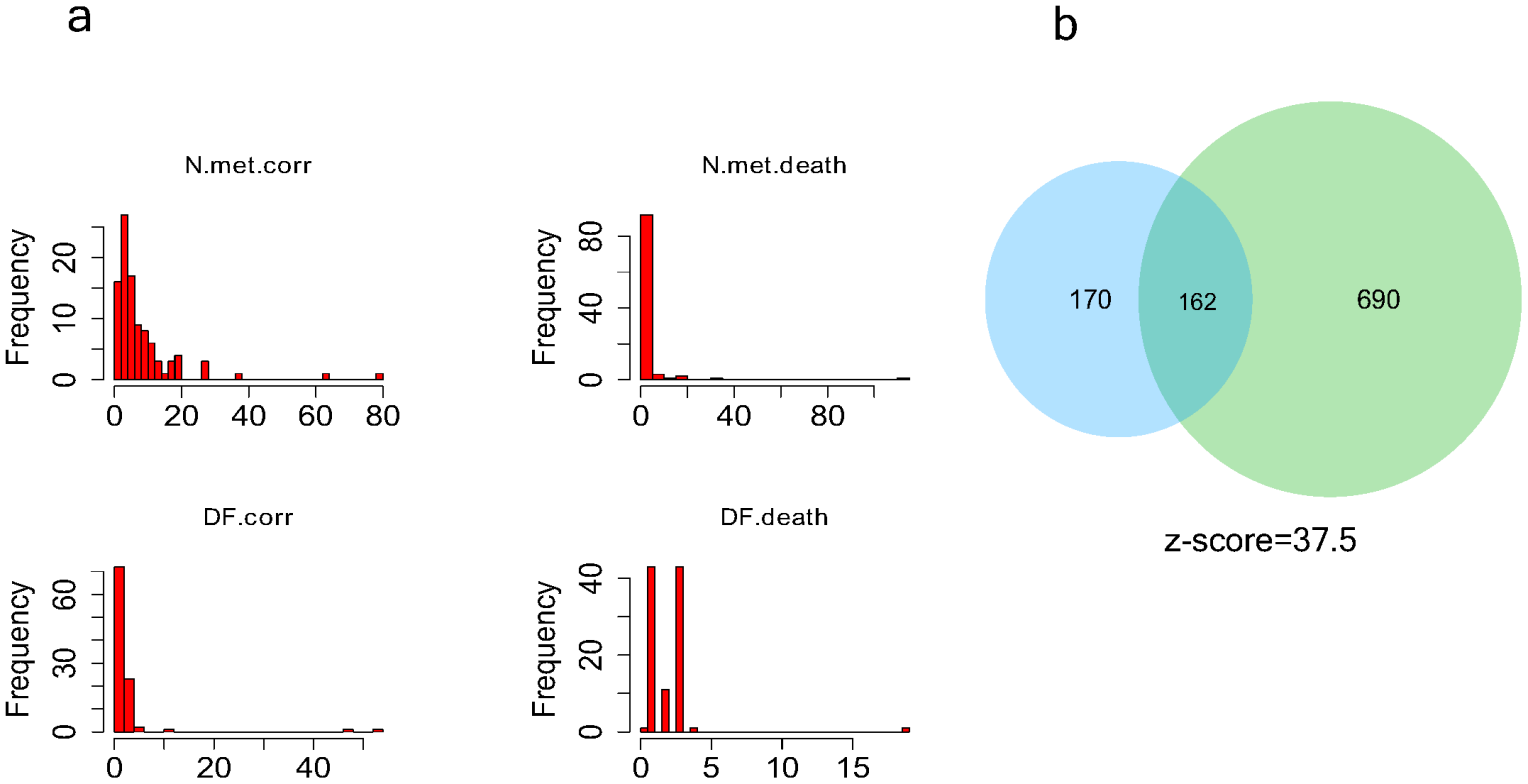
Panel a represents how the number of metabolites and the robustness of each metabolic sub-network are distributed (for both the growth correlated sub-networks and the mortality correlated sub-networks). Panel b shows the overlap between metabolites in the growth correlated sub-networks and the mortality correlated sub-networks.

The growth correlated metabolic sub-networks can be used for the identification of suitable anti-cancer drug targets. A common method for drug design is based on mimicking the chemical structure of a metabolite in order to use competitive inhibition of metabolic enzymes to decrease the metabolic flux in a particular pathway. The structure of the growth-correlated sub-networks can be used as a guide for the design of new drugs. First of all it is possible to identify metabolites that are present only in the growth correlated metabolic sub-networks, in order to avoid interferences with other metabolic pathways. Figure 7 shows the histogram of the number of unique metabolites in each sub-network, for the growth correlated sub-networks and for the colon cancer mortality associated sub-networks respectively.

Each of the metabolites unique to a sub-network, if it is assumed to be in steady state, can be used to define a mass balance equation that relates the fluxes of the reactions producing the metabolite and the fluxes of the reactions consuming it. The number degrees of freedom of the system of equations that results for each sub-network can be used as a measurement of its robustness. For example, a sub-network formed by a set of reactions in series has a robustness of 1 while a sub-network formed by a set of reactions operating in parallel would have as many degrees of freedom as reactions. Disabling a single reaction, in the first case would stop the flux through the whole pathway, while in the second case it would only have a minor impact. This can be a criterion to decide what metabolic sub-networks are more fragile and therefore constitute better targets for drugs. Figure 7 shows the robustness histogram for the growth correlated sub-networks and for the colon cancer mortality associated sub-networks respectively.

Finally, it is also possible to compute the degrees of freedom of a sub-network after blocking all the reactions consuming and producing a particular metabolite (which mimics the effects of a drug showing competitive inhibition against the enzymes processing the metabolite). If the remaining degrees of freedom are zero, the metabolite can be considered to be essential for the network operation and a possible scaffold for drug design.

As a conclusion we can say that in this paper we show how human genome-scale biological networks, and in particular genome-scale metabolic networks, can be used as analysis scaffoldss of gene-expression data, in order to provide deeper insights into the molecular mechanisms associated to cancer cell proliferation. This kind of analysis has been made possible thanks to the recent availability of high quality genome-scale metabolic networks with comprehensive gene annotations. In particular the HMR database contains 3668 metabolic genes, allowing an almost full mapping of transcriptional changes of genes into metabolic reactions.

This has led to the identification of possible regulatory mechanisms controlling cancer proliferation. We also identified over 100 growth related metabolic sub-networks, which can be seen as an atlas of metabolic pathways whose complexity goes well beyond the metabolic features typically associated to cancer^1^,^2^.

Several of the identified metabolic processes had been already shown to be relevant in particular cancers. Our analysis allows putting these processes in a broader perspective and reveals interactions between them, such as the simultaneous synthesis and degradation of fatty acids and the synthesis of carnitine.

Based on the analysis of gene expression and observations of the secretion pattern of the NCI-60 cell panel^7^, we have hypothesized that an important source of ATP in proliferating cancer cells is obtained from the shuttling of reducing power contained in the cytosolic NADPH, to the mitochondrion. This involves an apparently futile cycle of fatty acid biosynthesis and degradation and implies a key role of L-carnitine and the CPT1C transporter.

A very important question is the extrapolability of the results obtained from the contextualized analysis of the gene expression patterns of the NCI-60 panel, to in vivo tumors. In order to address this point we have used the differentially expressed genes between two groups of colon cancer patients to identify metabolic sub-networks whose activity has a negative impact on patient survival. The analysis revealed a substantial overlap between the sub-networks identified in both analyses, which suggests that the same metabolic sub-networks involved in sustaining higher proliferation rates in-vitro are also more active in more aggressive in-vivo tumors. Comparing the sub-networks at a metabolite level we obtain a very statistically significant overlap.

We also suggested some criteria to identify suitable drug targets against cancer proliferation, which are based in the topological structure of the metabolic sub-networks, which determines their robustness.

## Methods

The normalized HG-U133 Plus 2.0 and the HG-U95 microarrays were obtained from Cell-Miner (http://discover.nci.nih.gov). The specific growth rates of each cell line are calculated from the doubling times reported at Cell-Miner and they are reported in the supplementary file S10. The mapping between different gene IDs has been don using Clone/Gene ID converter^52^. The transcription factor enrichment analysis was done using Pscan^24^. The algorithms to identify metabolic sub-networks have been implemented as MATLAB functions and are available upon request. The data analysis and computational methods are described in detail in the supplementary methods (supplementary methods file).

## Author Contributions

S.B. designed the project, performed the analysis and wrote the manuscript, A.F. prepared the figures and contributed to the improvement of the manuscript.

## Supplementary Material

Supplementary Information function Clusters

Supplementary Information function expand

Supplementary Information function Graphstructure

Supplementary Information function multipleset

Supplementary Information S1

Supplementary Information S2

Supplementary Information S3

Supplementary Information S4

Supplementary Information S5

Supplementary Information S6

Supplementary Information S7

Supplementary Information S8

Supplementary Information S9

Supplementary Information S10

Supplementary Information Supplementary methods
