# Supplementary material for: Metabolic and protein interaction sub-networks controlling the proliferation rate of cancer cells and their impact on patient survival
Source: Sci Rep. 2013 Oct 24;3:3041. doi: 10.1038/srep03041 (PMC3807112; doi:10.1038/srep03041)
Supplement: Supplementary Information — Supplementary methods [file srep03041-s15.doc]

**Metabolic sub-networks controlling the proliferation rate of cancer cells and their impact on patient survival.**

**Amir Feizi, Sergio Bordel**

Department of Chemical and Biological Engineering, Kemivägen 10, Chalmers University of Technology, SE412 96 Gothenburg, Sweden.

**SUPPLEMENTARY METHODS**

1. **Multiple testing and calculation of the posterior probabilities of the null hypothesis (the h-values).**

For any statistical test that involves computing the probability of a statistic θ to be higher or equal than an observed value Θ under a null hypothesis H_0_, this probability Pr(θ$\geq$Θ|H_0_) is known as p-value. Applying the Bayes theorem we can obtain the probability of the null hypothesis when the statistic θ is higher or equal than the observed value Θ.

*Pr(H_0_|θ$\geq$Θ)=Pr(H_0_) Pr(θ$\geq$Θ|H_0_)/ Pr(θ$\geq$Θ)* [1]

By definition, the experiment i belongs to the region defined by θ$\geq$Θ_i_, therefore, the posterior probability of the null hypothesis for this experiment is:

*Pr(H_0i_)=Pr(H_0_) p_i_/ Pr(θ$\geq$Θ_i_)* [2]

Pr(H_0i_) will be written just as h_i_ and will be referred as h-value. If the experiments have been ranked in increasing order of p-values, the quantity Pr(θ$\geq$Θ_i_) can be estimated by the ratio i/m, being m the total number of experiments. A value of the prior probability Pr(H_0_) equal to one will be used because in practice, for all the experiments, the value Θ_i_ is transformed into a p-value p_i_ under the assumption that the null hypothesis is true.

The h-values of an experiment set can be just obtained using the following expression:

$h_{i}=\frac{m}{i}p_{i}$ [3]

In the correlation analysis, the h-values calculated from the distribution of p-values are just the probabilities of each probe not being correlated with the growth rate. These values can be used to obtain the false discovery rate committed when a set of *k* probes are considered to be correlated with the growth rate:

$q_{k}=\frac{\sum_{i=1}^{k} h_{i}}{k}$ [8]

1. **Definition of the set of top-correlated genes.**

The transcription factor analysis and the protein interaction sub-network analysis are based on the definition of a set of top correlated genes. Using equation 8 we can set the false discovery rate to a desired value and get the set of probes that correspond to the desired false discovery rate for both the HG-U133 Plus 2.0 and the HG-U95 microarrays. If a gene appears to have significantly correlated probes in both microarrays, it is then added to the set of top correlated genes.

1. **Assigning an h-value to each metabolic reaction.**

Once the h-values have been obtained for each probe, we use the reaction gene associations contained in the genome-scale metabolic networks in order to infer an h-value for each reaction.

In this case we start by pulling all the probes from the HG-U133 Plus 2.0 and the HG-U95 microarrays in a single set and obtaining h-values for each probe. If a gene has several proves we keep the lowest h-value. Then, for each metabolic reaction we define a new h-value which is the product of the h-values of all the genes associated to this reaction.

$h_{reaction}=\prod_{genes} h_{gene}$ [9]

1. **Measurement of similarity between reactions in a metabolic network.**

In other approaches to metabolic sub-network analysis, two reactions are considered to be contiguous if they share a metabolite. In this way, the structure of the metabolic networks, which topologically are Petri nets, is transformed into a graph. In other words, a Petri net is mapped into a structure with a different topology. Here, instead of the adjacency criterion, we use the Petri net structure of the metabolic network to define a degree of similarity between any pair of metabolic reactions in the network. This degree of similarity is the cosinus of the angle formed between the projections of two reactions into the null space of the stoichiometric matrix of the network. In this way, reactions whose fluxes are fully stoichiometrically coupled, such as those in a linear pathway, will be assigned a similarity of 1.

We provide here an example illustrating this principle. The network depicted in figure S1, can be represented by its stoichiometric matrix S, which contains the stoichiometric coefficients of each of its internal metabolites in each of the reactions of the network. Each metabolite corresponds to a row in the matrix and each reaction corresponds to a column. Every steady state flux distribution in a metabolic network belongs to the null space of its stoichiometric matrix.


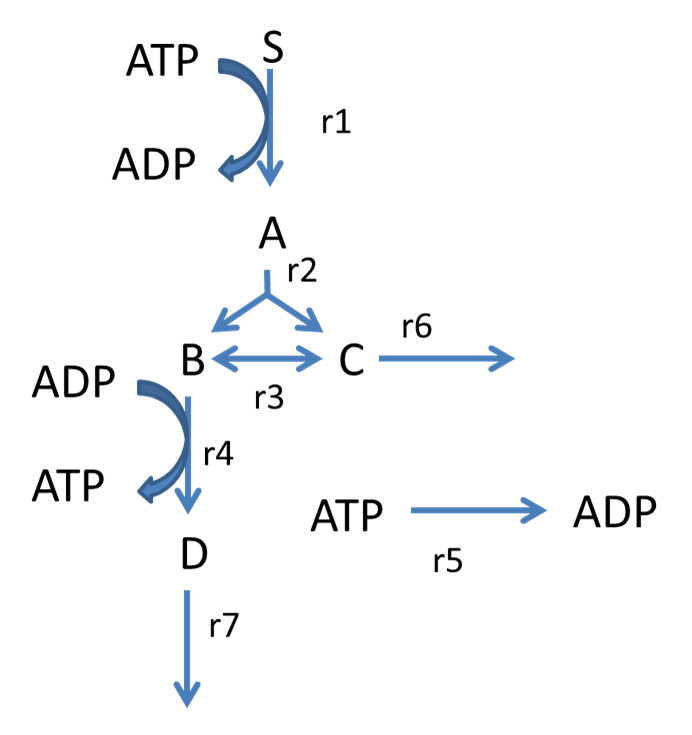


*Figure S1. Metabolic network in which the compounds A, B, C, D, ATP and ADP are internal metabolites.*

The stoichiometric matrix representing the network is the following one. The row representing ADP has been removed because it depends linearly of the row representing ATP.

$$S=\left(\begin{matrix} \text{ 1 -1 0 0 0 0 0} \\ \text{ 0 1 -1 -1 0 0 0} \\ \text{ 0 0 1 0 0 -1 0} \\ \text{ 0 0 0 1 0 0 -1} \\ \text{ -1 0 0 1 -1 0 0} \end{matrix}\right)$$

The null space of the stoichiometric matrix has two dimensions and the two columns of the matrix K form an orthonormal basis of this null space.

$$K=\left(\begin{matrix} \text{ 0.3960 0.3946} \\ \text{ 0.3960 0.3946} \\ \text{ 0.4741 -0.1590} \\ \text{ -0.0780 0.5535} \\ \text{ -0.4741 0.1590} \\ \text{ 0.4741 -0.1590} \\ \text{ -0.0780 0.5535} \end{matrix}\right)$$

The rows of the matrix K represent the projections of each of the reactions on the null space. The cosinuses of the angles between each pair of reactions are represented in the following matrix. We can see that the reactions that are stoichiometrically coupled (such as r1 and r2 or r3 and r6) have zero angles between each other and that reactions with fluxes fully anti-correlated have angles of 180 degrees.

$$Cos=\left(\begin{matrix} \text{ 1.0000 1.0000 0.4472 0.6000 -0.4472 0.4472 0.6000} \\ \text{ 1.0000 1.0000 0.4472 0.6000 -0.4472 0.4472 0.6000} \\ \text{ 0.4472 0.4472 1.0000 -0.4472 -1.0000 1.0000 -0.4472} \\ \text{ 0.6000 0.6000 -0.4472 1.0000 0.4472 -0.4472 1.0000} \\ \text{ -0.4472 -0.4472 -1.0000 0.4472 1.0000 -1.0000 0.4472} \\ \text{ 0.4472 0.4472 1.0000 -0.4472 -1.0000 1.0000 -0.4472} \\ \text{ 0.6000 0.6000 -0.4472 1.0000 0.4472 -0.4472 1.0000} \end{matrix}\right)$$

For the analyisis of genome-scale models, the reversible reactions have been duplicated (transformed into two reactions with opposed directions).


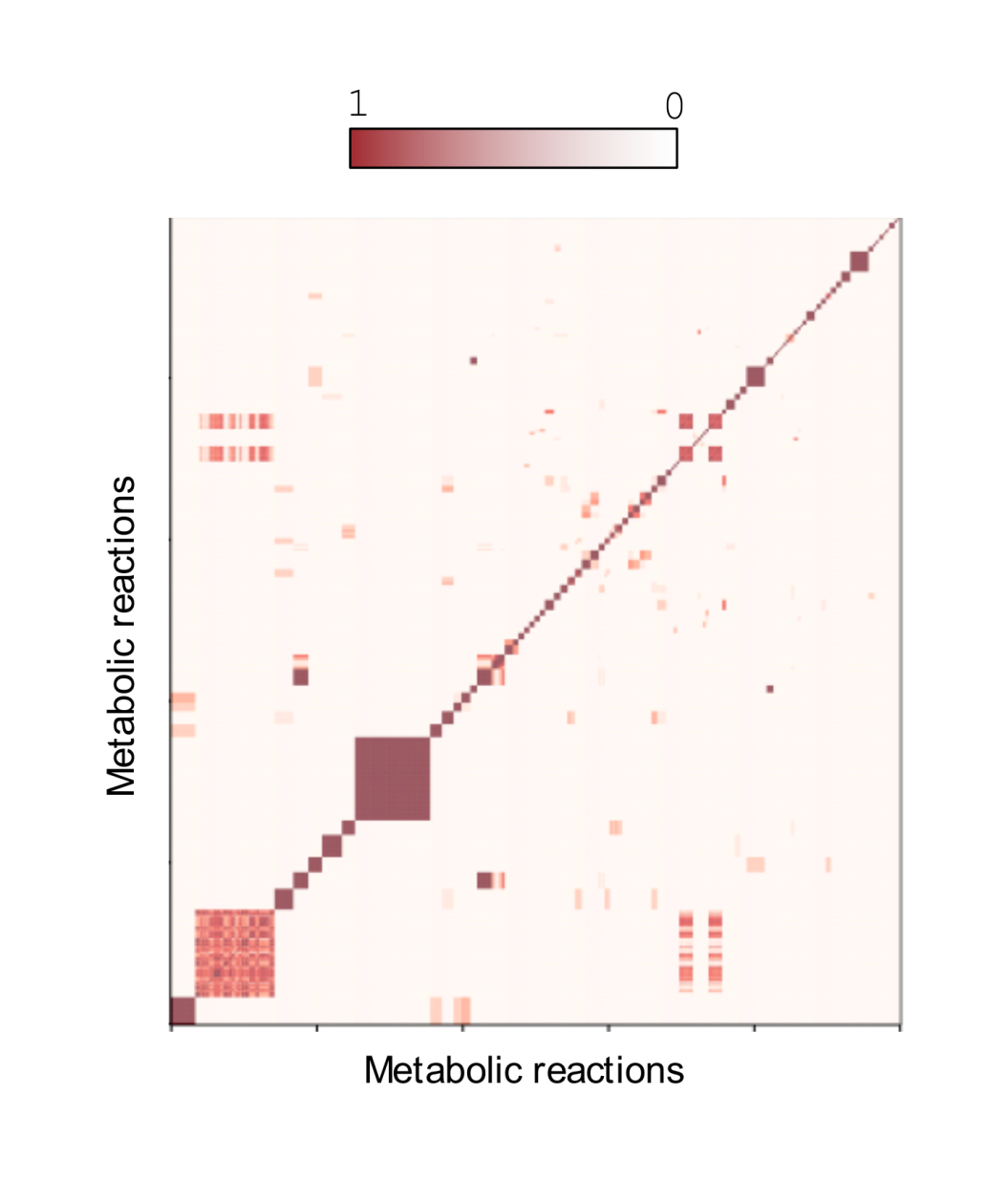


*Figure S2. Heat-map that represent the degree of similarity between the reactions in the top 100 metabolic sub-networks identified using the HMR database. It can be appreciated how the reactions form distinct clusters.*

1. **Identification of metabolic sub-networks.**

A matrix that combines the degree of similarity between reactions and the statistical significance of its correlation with the growth rate is defined in the following way:

$m_{ij}=\left(1-h_{i}\right)Cos\theta_{ij}\left(1-h_{j}\right)$ [10]

Two reactions are defined as connected if the resulting score is higher than a threshold that we have set to be 0.7. In this way we can find connected sub-networks of reactions with the characteristic of being topologically similar and showing some degree of positive correlation with the growth rate. These sub-networks are reported in the supplementary files S3, S5 and S7.

1. **Expanded metabolic sub-networks.**

The previous analysis can result in metabolic sub-networks with gaps. For example the keratan sulfate biosynthesis involves 37 steps among which 25 are growth correlated and appear in the metabolic sub-network. In order to get metabolic sub-networks without gaps, an algorithm which adds automatically the remaining 12 reactions to the network has been developed. The algorithm starts by computing the cosinus between every pair of reactions in the sub-network. Then for every reaction in the model that is outside the sub-network we compute its angle with each of the reactions in the pair. Let reactions i and j be in the network and k a reaction outside the network. If the following relationships are satisfied for every pair *i-j* in the network, reaction k is added to the expanded sub-network:

$Cos\theta_{ij}\leq Cos\theta_{ik}$ $Cos\theta_{ij}\leq Cos\theta_{jk}$ [11]

In the example of the keratan sulfate pathway the cosinus between every pair of reactions in the network is 1, therefore every other reaction with an angle of cosinus of 1 with any of the reactions in the network will be added to the expanded network. The expanded networks are reported in the files S4, S6 and S8.

1. **Network robustness and drug targets.**

We have defined the robustness of a sub-network as its number of degrees of freedom. For example the sub-network depicted in the figure S1 has a robustness of 2.

The effect of blocking all the reactions processing a metabolite can be measured by the number of degrees of freedom left after removing these reactions from the network. In the mentioned example all the metabolites would result in no degrees of freedom left to the network, so all of them could be equally valid as drug targets.

1. **Comparison at the metabolite level between growth-correlation and patient mortality.**

A good way to compare the results of the two types of analysis is to assess the degree of overlap between the metabolites unique to the top 100 sub-networks identified in each case. In the growth rate correlated sub-networks there are 852 metabolites that appear only within these sub-networks. In the case of the mortality correlated sub-networks there are 332 exclusive metabolites, 162 of these metabolites are in the intersection of both metabolite sets. The total number of metabolites in the model is 5552. This means that 15% of the total metabolites in the model are in growth-correlated metabolic sub-networks. If we take 332 random metabolites the number of expected metabolites belonging to the growth-correlated sub-networks can be approached by a normal distribution of average 51 (obtained multiplying the number of samples by the fraction 0.15) and standard deviation 2.96 (obtained multiplying the number of samples by the fraction 0.15 and 1-0.15 and taking the square root). The resulting Z-score is (162-51)/2.96=37.5. This correspond to a p-value so small that it cannot be computed exactly using MATLAB, the overlap between the growth-associated sub-networks and the mortality-associated sub-networks is strongly significant.

**MATLAB codes and tutorial**

We present here 4 MATLAB functions that we have used for the data analysis.

The function multipletest computes the h-values from a list of p-values.

h=multipletest(pval);

The function Graphstructure uses as an input a genome scale metabolic model (model), a list of statistical scores for each reaction in the model (reac), and the pre-computed kernel of the stoichiometric matrix (Khmr). Computing this kernel can take a long time for a big system, therefore is convenient to do it once and store it. The output of the function is the adjacency matrix of a graph (M) and the indexes (ids) of each metabolic reaction contained in the graph.

(M,ids)=Graphstructure(Khmr,reac,model);

The function Clusters, uses the output of the function Graphstructure to identify metabolic clusters. Each cluster is characterized by the indexes of the reactions that constitute the metabolic sub-network. It gives 100 networks as default, but this can be changed.

Clust=Clusters(M,ids);

The function cexpand takes as an input the identified clusters and adds to them new reactions in order to provide connected sub-networks.

Clustex=expand(Clust,Khmr);

function h=multipletest(pv);

y=1;

for i=1:numel(pv);

[u v]=min(pv);

f(i)=i/numel(pv);

alf(i)=u;

pv(v)=1.1;

Ra(i)=alf(i)/f(i);

bf(v)=Ra(i);

nfp(i)=sum(Ra);

FDR(i)=nfp(i)/i;

y=y*(1-Ra(i));

FWER(i)=1-y;

end

h=bf';

end

function [M,ind]=Graphstructure(Khmr,reac,modelhmr);

[m n]=size(Khmr);

Rhmr=[];

Rhhmr=[];

Rrxnhmr=[];

for i=1:m;

if reac(i)<0.5;

if modelhmr.rev(i)==1;

Rhmr=[Rhmr;Khmr(i,:);-Khmr(i,:)];

Rhhmr=[Rhhmr;reac(i);reac(i)];

Rrxnhmr=[Rrxnhmr;i;i];

else

Rhmr=[Rhmr;Khmr(i,:)];

Rhhmr=[Rhhmr;reac(i)];

Rrxnhmr=[Rrxnhmr;i];

end

end

end

ind=Rrxnhmr;

N=Rhmr*Rhmr';

[a b]=size(N);

Norma=zeros(a,b);

for i=1:numel(N(1,:));

Norma(i,i)=N(i,i)^-0.5;

end

H=zeros(a,b);

for i=1:numel(N(1,:));

H(i,i)=1-Rhhmr(i);

end

NN=Norma*N*Norma;

for i=1:numel(N(1,:));

NN(i,i)=0;

end

M=H*NN*H;

end

function clust=Clusters(M,ids);

mmax=max(M);

eli=[];

for i=1:numel(mmax);

if mmax(i)<0.7;

eli=[eli i];

end

end

M(eli,:)=[];

M(:,eli)=[];

ids(eli)=[];

for i=1:numel(M(1,:));

for j=1:numel(M(1,:));

if M(i,j)>0.7;

M(i,j)=1;

else

M(i,j)=0;

end

end

end

for j=1:100;

suma=sum(M);

[u v]=max(suma);

vec=zeros(numel(M(:,1)),1);

vec(v)=1;

veco=vec+M*vec;

for i=1:numel(veco);

if veco(i)>1;

veco(i)=1;

end

end

while sum(vec)<sum(veco);

vec=veco;

veco=vec+M*vec;

for i=1:numel(veco);

if veco(i)>1;

veco(i)=1;

end

end

end

clust{j}=[];

eli=[];

for i=1:numel(veco);

if veco(i)==1;

clust{j}=[clust{j} ids(i)];

eli=[eli i];

end

end

M(eli,:)=[];

M(:,eli)=[];

ids(eli)=[];

end

end

function Gex=expand(clusthmr,Khmr);

norma=zeros(numel(Khmr(:,1)),numel(Khmr(:,1)));

for j=1:numel(Khmr(:,1));

norma(j,j)=1/(Khmr(j,:)*Khmr(j,:)')^0.5;

end

for k=1:numel(clusthmr);

nor=zeros(numel(clusthmr{k}),numel(clusthmr{k}));

for i=1:numel(clusthmr{k});

nor(i,i)=1/(Khmr(clusthmr{k}(i),:)*Khmr(clusthmr{k}(i),:)')^0.5;

end

Ang1=nor*Khmr(clusthmr{k},:)*Khmr(clusthmr{k},:)'*nor;

Ang1=abs(Ang1);

Ang2=nor*Khmr(clusthmr{k},:)*Khmr'*norma;

Ang2=abs(Ang2);

mang=min(Ang1);

Gex{k}=[];

for j=1:numel(Ang2(1,:));

num=0;

for i=1:numel(mang);

if mang(i)<Ang2(i,j)+.000001;

num=num+1;

end

end

if num==numel(mang);

Gex{k}=[Gex{k} j];

end

end

end

end
